# Risk-Association of *DNMT1* Gene Polymorphisms with Coronary Artery Disease in Chinese Han Population

**DOI:** 10.3390/ijms151222694

**Published:** 2014-12-08

**Authors:** Chunyan Peng, Qianyun Deng, Zuhua Li, Chenling Xiong, Cong Li, Fang Zheng

**Affiliations:** 1 Center for Gene Diagnosis, Zhongnan Hospital of Wuhan University, Donghu Rd 169, Wuhan 430071, China; E-Mails: pengsparrow919@163.com (C.P.); dengqianyun1991@163.com (Q.D.); manbuyunhailzh@126.com (Z.L); xchenling@yahoo.com (C.X.); 2 Department of Molecular Pathology, International Agency for Research on Cancer, 150 Cours Albert Thomas, Lyon 69372, France; E-Mail: lic@fellows.iarc.fr

**Keywords:** DNA methylation, coronary heart disease, *DNMT1*, SNP

## Abstract

Recently, a significant epigenetic component in the pathogenesis of Coronary Artery Disease (CAD) has been realized. Here, we evaluated the possible association of candidate Single Nucleotide Polymorphisms (SNPs) in the epigenetic-regulatory gene, DNA methyltransferase 1 (*DNMT1*), with CAD in Chinese Han population. Five tag SNPs (rs16999593, rs2336691, rs2228611, rs4804494, rs7253062) were analyzed by High Resolution Melt (HRM) method in 476 CAD patients and 478 controls. Overall, there were significant differences in the genotype and allele distributions of rs2228611 and rs2336691, between patients and controls. The minor A allele of rs2228611 was associated with a lower risk of CAD (*p* = 0.034); modest effect in the additive analysis but also marginal significance was found in the recessive model [OR_additive_ = 0.404 (0.184, 0.884), *p* = 0.023 and OR_recessive_ = 0.452 (0.213, 0.963), *p* = 0.040] after adjusting for confounders. While the rs2336691 A allele were associated with a higher risk of developing CAD (*p* = 0.037); borderline significant association in both additive and dominant models [OR_additive_ = 1.632 (1.030, 2.583), *p* = 0.037 and OR_dominant_ = 1.599 (1.020, 2.507), *p* = 0.040]. In conclusion, these data provide the first evidence that occurrence of CAD may be moderated by genetic variation in the gene involved in the epigenetic machinery.

## 1. Introduction

DNA methylation is an important cellular mechanism that modulates gene expression associated with aging, inflammation and atherosclerotic processes. Aberrant DNA methylation encompassing genome-wide hypomethylation and CpG island hypermethylation has been demonstrated in Coronary Artery Disease (CAD) [1,2]. Significant genomic hypomethylation was found both *in vivo* and *in vitro*, including aging smooth muscle cells, aortas of apolipoprotein E-knockout (Apo-E-) mice, as well as in advanced human atherosclerotic lesions [3,4,5]. On the other hand, hypermethylation of certain genes, including estrogen receptor 1 (ER-α), tissue factor pathway inhibitor 2 (TFPI-2), ATP-binding cassette sub-family A member 1 (ABCA1), occurred in vascular tissue or peripheral blood cells in CAD patient [6,7,8]. Recently, Guay *et al*. [9] found that peripheral blood DNA methylation at five key lipoprotein metabolism gene loci was significantly associated with the serum lipids level in familial hypercholesterolemia. Moreover, promoter hypermethylation of 11 mechanosensitive genes due to disturbed blood flow in carotid artery was also identified in mouse [10]. On the basis of these observations, we anticipated that perturbations of DNA methylation contributed to CAD risk variability.

DNA methylation is catalyzed by DNA methyltranferases. *DNMT1* is the major enzyme responsible for maintenance of the DNA methylation pattern and methylates newly biosynthesized DNA, and located on chromosome 19p13.2, including 40 exons and 39 introns [11]. Circumstantial evidences from endogenous *DNMT* *in situ* hybridization analysis supports the conclusion that increased *DNMT* expression is associated with increased cellular proliferation in atherosclerosis [5]. Recently, Jessilyn *et al.* [10] reported that disturbed blood flow epigenetically controls endothelial gene expression *in vitro* by regulating genome-wide DNA methylation patterns via a *DNMT1*-dependent mechanism in carotid artery. Therefore, it is reasonable to speculate that *DNMT1* gene might play a contributory role in the pathogenesis of CAD.

**Figure 1:**
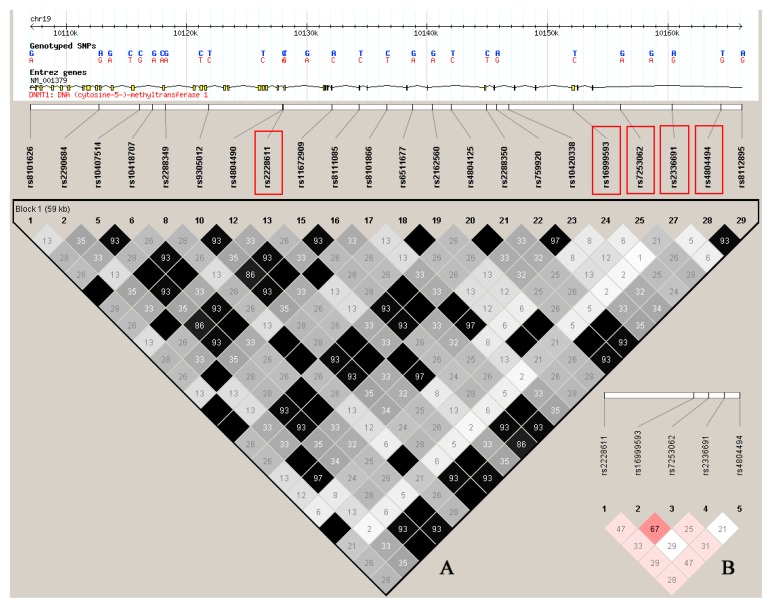
(**A**) Low-density (LD)-plot and haplotype blocks. Haplotype blocks generated by HaploView software (Broad Institute, Cambridge, MA, USA) based on the correlation between markers are illustrated in black. Five marker haplotype blocks highlighted in red were investigated in this research; (**B**) The schematic overview of linkage disequilibrium of the five studied SNPs (located on chromosome 19). The color scheme represents D’/LOD, while the numbers stand for *R*^2^.

Over the last few years, a lot of research on the gene expression and polymorphism causing CAD have been done that indicate the importance of identifying related SNPs in CAD. Notably, most DNA variants shown to be closely related to the cardiac disease risk were established within or near chromosome 9p21.3 [12], such as lipoprotein (a), coxsackie virus and adenovirus receptor, APOE. Given that there was no research to evaluate the epigenetic features of the human genome including DNA methylation related DNA variants. Therefore, we selected five tag SNPs of *DNMT1* gene located in distinct blocks of linkage disequilibrium (LD) according to HapMap CHB data (Figure 1) and identified two genetic variants (rs2228611 and rs2336691) on the *DNMT1* gene that were associated with increased risk of CAD.

## 2. Results

### 2.1. Clinical Characteristics of the Study Population

The clinical characteristics of the combined study cohort were detailed (Table 1). The proportion of male and the average age were both significantly higher in CAD patients than that of controls. Therefore, we applied Breslow-Day homogeneity tests to evaluate the effect of gender and age, and both did not show heterogeneity. Significantly higher ratios of hypertension, diabetes and hyperlipidemia were observed in the CAD patients compared to the controls. Since most patients with hypertension were on the antihypertensive therapy, the proportion of hypertensive patients was reported instead of blood pressure values. Plasma concentrations of total cholesterol (TC), low-density lipoprotein cholesterol (LDL-C) and high density lipoprotein cholesterol (HDL-C) were significantly lower, while total triglyceride (TG), fasting blood glucose (FBG) concentrations were significantly higher in the case group than those of the control group (Table 1).

**Table 1:** Baseline characteristics and risk factors of Coronary Artery Disease (CAD) cases and controls. * *p* < 0.05 for each clinical indicators, by comparing the control group with CAD group.

Clinical Data	Cases ( *n* = 476)	Control ( *n* = 478)
Age (years) *	60.61 ± 9.21	56.54 ± 9.59
Gender (M%) *	67.9	55.6
Hypertension (%)	68.1	0
DM (%) *	28.5	0
Hyperlipidemia (%) *	23.2	0
TC (mmol/L) ^1,^*	4.36 ± 1.07	4.44 ± 0.56
TG (mmol/L) *	1.57 ± 0.95	1.03 ± 0.30
HDL-C (mmol/L) *	1.15 ± 0.35	1.27 ± 0.22
LDL-C (mmol/L) ^1,^*	2.41 ± 0.85	2.63 ± 0.30
FBG (mmol/L) *	6.58 ± 2.51	4.84 ± 0.50

^1^ Most of the CAD patients with hyperlipidemia were under anti-hyperlipidemia therapy in the case group. DM: diabetes mellitus; TC: total cholesterol; TG: total triglyceride; HDL-C: High density lipoprotein cholesterol; LDL-C: low-density lipoprotein cholesterol; FBG: fasting blood glucos1e.

### 2.2. Allele Selection and Genotype Distributions

Five tag SNPs in *DNMT1* were chosen from five distinct blocks of LD according to HapMap CHB data (Figure 1), Table 2 shows detailed information regarding these SNPs. Most of the Minor Allele Frequencies (MAFs) observed in this study were close to those in dbSNP database (http://hapmap.org, phase2, HapMap-CHB, International HapMap project, Chinese National Human Genome Center, Beijing, China) except the minor A allele of rs2336691 was higher in our studied cohort (0.14) compared with the value from the HapMap database (0.06). No significant deviation from Hardy-Weinberg equilibrium (HWE) was observed for all tested SNPs both in the controls and the cases (*p* value > 0.05). Figure S1 shows the normalized melting curves and peaks of small amplicons.

**Table 2:** Characteristics of SNPs in *DNMT1* gene.

SNP	Position ^1^	Location	Minor Allele	Major Allele	Codon Change	MAF ^2^	MAF ^3^
rs2228611	10267077	Exon 18 region	A	G	Pro463Pro	0.32	0.35
rs16999593	10291181	Exon 4	C	T	His97Arg	0.17	0.19
rs7253062	10295124	Intron 1	A	G	-	0.26	0.29
rs2336691	10299392	Intron 1	A	G	-	0.06	0.14
rs4804494	10303449	Intron 1	G	T	-	0.40	0.37

^1^ Position in basepairs was derived from dbSNP Build 138 Variations of chromosome 19 (http://genome.ucsc.edu, University of California Santa Cruz, CA, USA); ^2^ MAFs (Minor Allele Frequency) were derived from Hapmap-CHB (http://hapmap.org, phase2, HapMap-CHB); ^3^ MAFs observed in this research (based on a total number of 954 participants).

Linkage disequilibrium was performed with Haploview software (Figure 1). The SNP linage disequilibrium patterns were assessed using *r*^2^ values. Based on the HapMap database, *R*^2^ value were less than 0.8 among the five SNPs, suggesting that they do not exist in linkage disequilibrium with each other.

### 2.3. Two SNPs Associated with CAD.

As shown in Table 3, there were significant differences in the genotype and allele distributions of rs2228611 and rs2336691, between patients and controls. The minor A allele of rs2228611 was associated with a lower risk of CAD (*p* = 0.034); greatest effect in the additive analysis but also marginal significance was found in the recessive model [*OR* = 0.404 (0.184, 0.884), *p* = 0.023 and *OR* = 0.452 (0.213, 0.963), *p* = 0.040] after adjusting for confounders (age, gender, hypertension, hyperlipidemia and diabetes mellitus). While the rs2336691 A allele were associated with a higher risk of CAD (*p* = 0.037); borderline significant association in both additive and dominant model [*OR* = 1.632 (1.030, 2.583), *p* = 0.037 and *OR* = 1.599 (1.020, 2.507), *p* = 0.040]. However, most of the statistically significant findings will be abolished if we apply correction of multiple testing (*p* < 0.01) in this study.

No association was identified between rs16999593, rs7253062, rs4804494 and CAD in this research after considering age, gender, hypertension, hyperlipidemia and diabetes mellitus as covariates (Table 3).

For the polymorphism rs2228611, no difference was identified between simple CAD cases and any groups with comorbidity (Table S1). CAD patients with diabetes have a marginal significant lower A allele frequencies of rs2336691 compared to non-diabetes CAD patients (*p* = 0.045) (Table S1).

### 2.4. Rs7253062 Associated with Hyperlipidemia

Multiple stepwise logistic regression analyses indicated that the polymorphism rs7253062 was significantly associated with CAD in the first unadjusted step in recessive model (Table 4): the observed *p* value without adjusting in the recessive model was 0.005 [*OR* = 1.711 (1.173, 2.495)]. The associations still significant after adjusted for confounders without hyperlipidemia [*OR* = 1.923 (1.070, 3.454), *p* = 0.029]; however, this relationship vanished [*OR* = 1.171 (0.549, 2.497), *p* = 0.682] after hyperlipidemia adding into adjusting options (Table 4).

### 2.5. Haplotype Analyses

Table 5 presents the haplotype frequencies (≥3% in either CAD patients or controls) of five examined polymorphisms in CAD patients and controls with the cumulative frequencies reaching 86.34% and 77.32% respectively. The most common haplotype A-T-G-G-T was comparable in frequencies between patients and controls (*p*_sim_ = 0.618), and was assigned as the reference group in risk estimates. Haplotypes G-T-A-A-T, which was remarkably overrepresented in patients, was associated with increased risk of developing CAD after considering age, gender, hypertension, hyperlipidemia and diabetes mellitus as covariates [*OR* = 2.097 (1.612, 4.863), *p* < 0.001].

**Table 3:** Risk estimated based on the distributions of genotype and allele frequency.

SNP	Genotype	Control (*n* = 478)	CAD (*n* = 476)	Allele OR (95% CI)	*p*	Additive OR (95% CI)	*Adj-p*	Dominant OR (95% CI)	*Adj-p*	Recessive OR (95% CI)	*Adj-p*
rs2228611	GG	192	221	*0.812 (0.669, 0.984)*	*0.034*	reference	-	reference	-	reference	-
	AG	212	195	-	-	0.796 (0.516, 1.228)	0.303	0.696 (0.460, 1.054)	0.087	-	-
	AA	74	60	-	-	*0.404 (0.184, 0.884)*	*0.023*	-	-	*0.452 (0.213, 0.963)*	*0.040*
rs16999593	TT	304	315	0.901 (0.705, 1.151)	0.404	reference	-	reference	-	reference	-
	CT	153	147	-	-	0.881 (0.562, 1.379)	0.578	0.900 (0.587, 1.380)	0.628	-	-
	CC	21	14	-	-	1.045 (0.391, 2.793)	0.930	-	-	1.090(0.412, 2.882)	0.863
rs7253062	GG	242	241	1.002 (0.815, 1.233)	0.981	reference	-	reference	-	reference	-
	AG	203	178	-	-	1.163 (0.755, 1.792)	0.494	1.177 (0.777, 1.782)	0.442	-	-
	AA	33	57	-	-	1.257 (0.573, 2.759)	0.568	-	-	1.171(0.549, 2.497)	0.682
rs2336691	GG	372	340	*1.320 (1.017, 1.714)*	*0.037*	reference	-	reference	-	reference	-
	AG	98	119	-	-	*1.632 (1.030, 2.583)*	*0.037*	*1.599 (1.020, 2.507)*	*0.040*	-	-
	AA	8	17	-	-	1.207 (0.258, 5.645)	0.811	-	-	1.063(0.228, 4.947)	0.938
rs4804494	TT	171	200	0.836 (0.685, 1.019)	0.076	reference	-	reference	-	reference	-
	GT	238	217	-	-	1.228 (0.778, 1.940)	0.378	1.195 (0.770, 1.854)	0.428	-	-
	GG	69	59	-	-	1.071 (0.545, 2.103)	0.842	-	-	0.946(0.513, 1.746)	0.860

*p*, *p* values derived from logistic regression of allele frequency; Adj-*p*, *p* values derived from logistic regression after adjustment for age, gender, hypertension, hyperlipidemia, diabetes mellitus. Significant different values between cases and controls (*p* < 0.05) were shown in bold and italic style.

**Table 4:** Risk estimated based on the multiple stepwise adjustment in rs7253062.

OR (95% CI), *p*	rs7253062 *
OR (95% CI), *p*^1^	1.711 (1.173, 2.495), 0.005
OR (95% CI), *p*^2^	1.388 (0.848, 2.274), 0.192
OR (95% CI), *p*^3^	1.923 (1.070, 3.454), 0.029
OR (95% CI), *p*^4^	1.198 (0.626, 2.291), 0.585
OR (95% CI), *p*^5^	1.171 (0.549, 2.497), 0.682

^1^ observed value without adjustment; ^2^ value after adjusted for confounders without hypertension; ^3^ value after adjusted for confounders without hyperlipidemia; ^4^ value after adjusted for confounders without diabetes mellitus; ^5^ value after adjusted for confounders (age, gender, hypertension, hyperlipidemia, diabetes mellitus). * In recessive model.

**Table 5:** Haplotype frequencies of five SNPs examined in *DNMT1* gene between patients and controls and their risk prediction for CAD.

Haplotye ^1^	CAD Patients (%)	Controls (%)	*p*_sim_	OR (95% CI) *p*
A-T-G-G-T	20.19	18.57	0.618	Reference group
G-T-A-G-T	14.38	8.18	0.050	0.917 (0.653~1.288) 0.057
G-T-G-G-G	13.60	10.34	0.385	2.532 (1.465, 4.376) 0.391
G-T-G-G-T	10.43	13.11	0.503	1.014 (0.989, 1.039) 0.507
G-C-G-G-G	10.05	8.23	0.360	1.411 (0.700, 2.845) 0.357
***G-T-A-A-T***	***7.20***	***2.72***	***0.003***	***2.097 (1.612, 4.863) <0.001***
G-C-G-G-T	4.03	3.95	0.572	0.661 (0.286, 1.530) 0.567
A-T-A-G-T	3.71	5.44	0.408	1.343 (0.698~2.580) 0.387
A-T-G-G-G	2.75	6.78	0.145	0.343 (0.082, 1.443) 0.148

^1^ Allele in haplotype were presented in order of polymorphisms rs2228611, rs16999593, rs7253062, rs2336691 and rs4804494; OR (95% CI) *p*, *p* values were calculated after adjusting for age, gender, hypertension, hyperlipidemia and diabetes mellitus. The genotype which was significant different between cases and controls was shown in bold and italic style.

## 3. Discussion

In the present study, we sought to investigate the association of five SNPs on methylation-regulatory gene, *DNMT1*, with the risk of CAD in the Chinese Han population involving 954 individuals. To the best of our knowledge, this report so far is the first case-control study examining the susceptibility of *DNMT1* genetic variants to CAD.

One of the principal finding in this research is that the polymorphism rs2228611, which situates in the exon 18 of *DNMT1* and leads to synonymous variation, belonging to a linkage group consisting of more than six SNPs in the same vicinity, associated with increased risk of CAD under both additive and recessive models after adjusting for confounders. The frequencies of minor A allele in rs2228611 were significantly lower in CAD patients, indicating that the A allele might be a protective factor for CAD. Actually, previous research has shown that the polymorphism rs2228611 contributed to a range of human diseases: the G allele of rs2228611 was reported to be a risk factor of ovarian cancer in a polish population [13] and sporadic infiltrating ductal breast carcinoma among northern Chinese women [14]. Since the genetic variation in this locus did not result in amino acid changes, silent or synonymous mutations might be causal through influencing promoter activity and the conformation and stability of pre-mRNAs [15], or influence the translation efficiency and protein folding and function by changing the structure of the substrate site [16,17], and alternatively this variant may also be in LD with other yet unknown functional variants.

Our studies have also demonstrated an association of the polymorphism rs2336691 with CAD, which is located in the intron 1 of *DNMT1*. According to the NCBI dbSNP data base (National Center for Biotechnology Information, Bethesda, MD, USA), MAF (minor allele frequency) of this locus in CHB population is very low (around 0.06); however, in this study, the frequency of minor A allele accounted for 14% (Table 2). As demonstrated in our single-locus analyses, these polymorphisms were significantly associated with the risk of developing CAD, under both additive and dominant model (Table 3). The frequency of the rare A allele in the rs2336691 was significantly higher in patients than controls (Table 3), indicating that rare A allele might be a potential risk factor for CAD. Moreover, when we were analyzing the associations between different pathological status and genotype distributions in the case cohort, we found that CAD patients with diabetes have a marginal significant lower A allele frequencies compared to non-diabetes CAD patients (*p* = 0.045) (Table S1). Further in haplotype analyses, G-T-A-A-T (alleles in order of rs2228611, rs16999593, rs7253062, rs2336691 and rs4804494), which harboring the rare A allele in rs2336691, was the only identified haplotype that have a significant association with CAD compared with reference A-T-G-G-T haplotype (Table 5). From a biological standpoint, it cannot exclude the possibility that the intronic rs2336691 might be functional given the potential regulatory effect of intronic locus on the stability of DNA molecule [18], or regulate gene transcription and expression by generating intronic miRNA [19], or might also be a genetic marker in linkage disequilibrium with other mutations or variations in regulatory regions of *DNMT1*.

In haplotype analyses, the rs7253062 provided another minor A allele to the G-T-A-A-T (alleles in order of rs2228611, rs16999593, rs7253062, rs2336691 and rs4804494) genotype, which contributes to the increasing risk of CAD (Table 5). However, in our single-locus analyses, rs7253062 was not associated with CAD after adjusting for confounders (age, gender, hypertension, hyperlipidemia, diabetes mellitus) (Table 3). During multiple stepwise logistic regression analyzing, we found that this SNP was significant associated with CAD before adjustment or after adjusting for particular factors (Table 4). Hyperlipidemia adjustment reversed the significant association of rs7253062 between CAD cases and controls in the recessive model and we got significant difference in the distributions of the SNP between controls and CAD patients with hyperlipidemia (*p* = 0.021) (Table S2), suggesting that the rs7253062-CAD association might be mediated through blood lipids level alteration.

## 4. Experimental Section

### 4.1. Subjects

The study subjects (*n* = 954) consisted of 476 CAD patients and 478 matched (ethnic group, gender, age) CAD-free individuals, who had been part of a larger population examined in our previous work [20] (Table 1). All samples were collected from Zhongnan Hospital of Wuhan University and Asia Heart Hospital between January and June of 2011 in Wuhan, China, and they were randomly selected and assigned to discovery and replication panels.

All CAD patients underwent diagnosis of coronary angiography; CAD was defined as ≥50% stenosis in one or more major coronary artery, medical records of percutaneous coronary angioplasty, coronary artery bypass graft or myocardial infarction. Patients with myocardial bridge, congenital heart disease or other types of atherosclerotic lesions were excluded. Hypertension was defined as a clinical blood pressure of ≥140/90 mmHg or history of medication. Diabetes mellitus refers to type 2 diabetes. All study participants are of the ethnic Han origin by self-report. Relevant data were also collected from all the participants by direct interviews or from medical-case files including age, gender, lipid profiles, history of hypertension, diabetes mellitus and dyslipidemia. Clinical examinations were carried out using rest electrocardiograms (ECG) for the control participants. All control participants showed no signs of CAD, hypertension, diabetes mellitus or dyslipidemia based on the ECG results and medical-case files at the time of enrollment. Blood pressure was measured using a standard mercury sphygmomanometer on the left arm after 5 min rest in the sit position. Fasting blood glucose (FBG), total cholesterol (TC), total triglyceride (TG), high density lipoprotein cholesterol (HDL-C), low-density lipoprotein cholesterol (LDL-C) were analyzed by standard techniques, which were employed by the Core Laboratories in Zhongnan Hospital and Asia Heart Hospital. The study was approved by ethics committee of Zhongnan Hospital of Wuhan University and met the declaration of Helsinki.

### 4.2. SNPs Selection and Genotyping

Five SNPs showing the greatest variability in the *DNMT1* gene region were selected from the HapMap database with the criteria used in our SNP selection procedure [a minor allele frequency over 0.05 and tag SNPs with an *R*^2^ value <0.8]. Table 2 presents a description of the SNPs used. Blood samples were drawn from study participants and genomic DNA was isolated via the standard proteinase K digestion and phenol-chloroform extraction. SNPs were genotyped by high-resolution melting of small amplicons on LightScanner 96 instrument (Idaho Technology, Salt Lake City, ID, USA) [21]. Primer details and product lengths are shown in Table S3.

### 4.3. Statistical Analysis

Statistical analyses were performed with SPSS 18.0 for Windows. Normally distributed data were shown as the mean ± S.D. Allele and genotypic associations of SNPs with CAD were assessed using unconditional logistic regression (SPSS, version 18.0, Chicago, IL, USA) and Pearson’s 2 × 2 contingency table χ^2^ test. Deviation of allele frequency from Hardy-Weinberg equilibrium (HWE) was tested for all SNPs using the Haploview software. In addition, LD pattern between the five studied SNPs was tested also using Haploview. ORs and 95% confidence intervals (CIs) were estimated using unconditional logistic regression and the χ^2^ test (SPSS, version 18.0), and shown as [*OR* (95% CI), *p* =] in the passage. When the case-control samples were stratified, Breslow-Day tests were performed to analyze the homogeneity between ORs from each sub-group (SPSS, version 18.0). Multivariable logistic regression analysis was performed by incorporating age, gender, hypertension, hyperlipidemia and diabetes mellitus covariates (SPSS, version 18.0). *p* values less than 0.05 were considered statistically significant.

The haplotype frequencies of five examined polymorphisms in *DNMT1* gene were estimated as previously reported [22]. Only haplotype with frequency ≥3% was considered in haplotype analyses. The haplo.cc and haplo.glm program were employed to calculate the adjusted odds rations (ORs) and 95% confidence intervals (CIs) for each haplotype. *p*_sim_ was calculated based on randomly permuting the trait and covariates and then computing the haplotype score statistics. Programs were implemented in Haplo.Stats software (version 16.0, Mayo Clinic Division of Health Sciences Research, Rochester, NY, USA) operated in the R language (version 3.05, http://www.r-project.org/, The R Foundation, Vienna, Austria).

## 5. Conclusions

In mammals, DNA methylation plays a pivotal role in development, ageing and pathological process of certain disease. Exploration of DNA methylation- regulatory mechanism is important since the extent of methylation can be associated with genomic instability, increased mutation events or altered gene expression [23,24]. Extensive studies demonstrated that both genome-wide hypomethylation and CpG island hypermethylation were implicated in CAD. *DNMT1* as the major methylation enzyme plays a critical role in the alteration of DNA methylation pattern. Our research provided preliminary evidence that SNPs (rs2228611, rs2336691) on *DNMT1* were associated with CAD. Since increased *DNMT* expression was associated with increased cellular proliferation in atherosclerosis [5], we speculate that the *DNMT1* expression levels might be altered under different genotypes which then contributes to the pathological process of CAD. It is necessary to explore the *DNMT1* expression levels in different genotypes in the further study to verify this hypothesis. In addition, neither of these two polymorphisms is a functional variant, and their association with CAD may be due to LD with one or more functional polymorphisms of *DNMT1*. There were limitations in this study. First, most of the adjusted *p* values in this research is near the boundary line (0.05), therefore the results need to be confirmed in the further studies with large sample size and in different ethnic populations; Second, the retrospective design of this study has inherent drawbacks and precludes causal inferences; Third, we only focused on the five tag-SNPs in *DNMT1* gene, and it is encouraged to examine other polymorphisms on the epigenetic-regulatory pathway, for example, *DNMT3A*, *DNMT3B*, histone deacetylase *et cetera*. Besides that, further research on the functions of polymorphism rs2228611 and rs2336691 is also needed to clarify the precise role of DNMT1 in the pathogenesis of CAD.

## Supplementary Materials

Supplementary materials can be found at http://www.mdpi.com/1422-0067/15/12/22694/s1.
